# Lighter and Simpler Design Paradigm for Widespread Use of Ankle Exosuits Based on Bio-Inspired Patterns

**DOI:** 10.3390/biomimetics7040148

**Published:** 2022-09-29

**Authors:** Sungjin Park, Junyoung Moon, June il Park, Jaewook Ryu, Kimoon Nam, Jaeha Yang, Giuk Lee

**Affiliations:** Department of Mechanical Engineering, Chung-Ang University, Seoul 06974, Korea

**Keywords:** soft wearable robot, ankle exosuit, bio-inspired pattern, wearability, gastrocnemius

## Abstract

Soft wearable robots are attracting immense attention owing to their high usability and wearability. In particular, studies on soft exosuits have achieved remarkable progress. Walking is one of the most basic human actions in daily life. During walking, the ankle joint has considerable influence. Therefore, an exosuit design paradigm having a light and simple structure was developed with the goal of fabricating a soft exosuit that supports the ankle. The new exosuit matches the performance of existing exosuits while being as comfortable as everyday wear. A walking test through a combination with a mobile actuator system, which can maximize these advantages, was also conducted. The combination with the mobile system demonstrates the potential of using the new ankle exosuit as inner wear that maximizes the advantages of a lighter and simpler design. The exosuit design paradigm could serve as an effective guideline for manufacturing assistive exosuits for various body parts in the future.

## 1. Introduction

**Figure 1:**
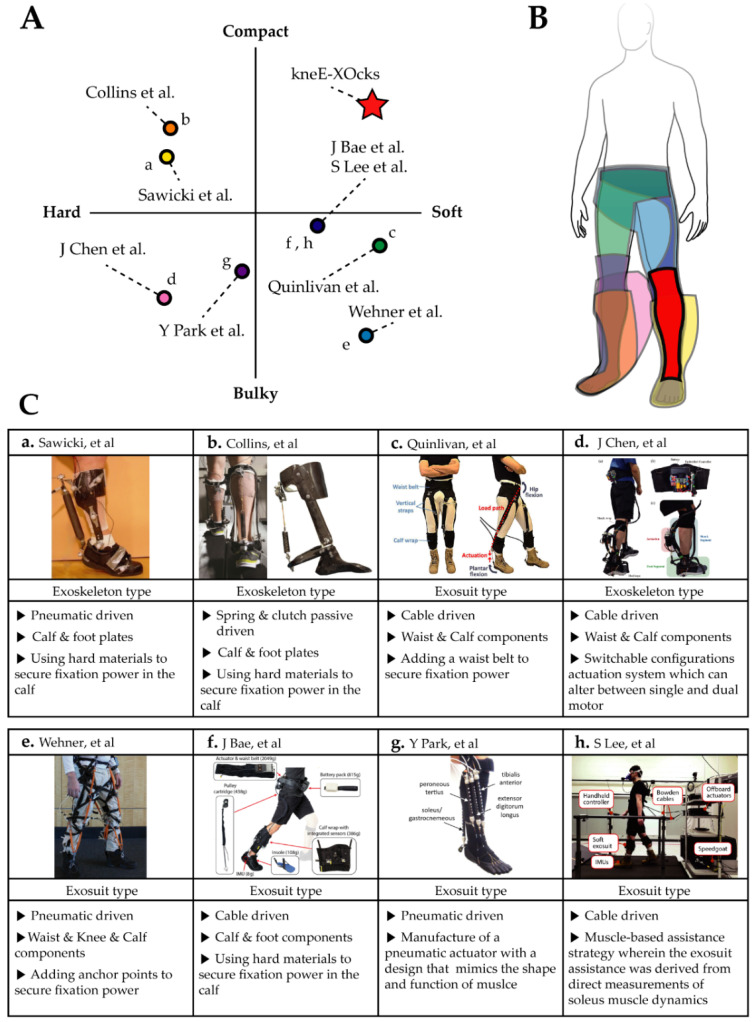
(**A**) Distribution chart of the degree of rigidity and volume of current wearable ankle robots. This image confirms the need for a new ankle robot that is soft and compact like kneE-XOcks. (**B**) Compact design of kneE-XOcks compared to existing wearable ankle robots. This can be confirmed through the illustration comparing the volume of kneE-XOcks and current wearable ankle robots. (**C**) Table analyzing and classifying the characteristics of current wearable ankle robots. Compared to existing wearable ankle robots, kneE-XOcks show a high auxiliary effect; consequently, they require a hard plate or additional fixing point in many parts [3,4,5,8,13,14,15,16,17].

Walking is the most basic movement in human life. People have always been interested in developing devices that can improve their walking abilities [1]. This has led to the development of various wearable devices that can increase the wearer’s walking efficiency. Over the past few decades, several researchers have developed various wearable devices to improve the walking efficiency; these studies have yielded notable results. As the ankle, in particular, yields the highest positive mechanical power output among all human joints [2], many researchers initially targeted their efforts toward assist devices tailored for the ankle joint. As shown in Figure 1C(a) [3], pneumatic exoskeletons used to assist the ankle achieved 6% metabolic cost reductions during loaded walking. Collins et al. [4] achieved 7.2% metabolic cost reduction during walking by using an unpowered exoskeleton adopted to assist the ankle (Figure 1C(b)). Quinlivan et al. [5] achieved metabolic cost reductions of approximately 23% with the gait assisted by a multiarticular soft exosuit (Figure 1C(c)). Recently, various research groups presented data that justified metabolic cost reductions, improved the wearer’s walking stability, and prevented injury [6]. Recently, research on the function of not only reducing metabolic cost but also increasing the wearer’s walking stability or preventing injury is being conducted [6]. Research has also been conducted to alleviate gait disturbances in people suffering the aftereffects of stroke through assistance-optimization of the preceding ankle exosuit [7]. Additionally, an ankle exosuit design has been proposed that converts the actuation system between a single motor and a dual motor (Figure 1C(d)) [8].

In future, these wearable assistive devices may be commonly used in everyday life. However, despite recent technological advancements, it is still not easy to use wearable devices. A majority of wearable devices are exoskeletons that consist of rigid structures attached onto the body in orientations parallel to the joints. The structure of the exoskeleton is heavy and bulky [9] and leads to increased discomfort and energy expenditure owing to the added inertia during walking, which makes it difficult to use the exoskeleton in everyday life [10]. Furthermore, if there are misalignments between the joint of the exoskeleton and human body, the exoskeleton can apply incorrect force to the joint and disrupt natural locomotion. Despite the advanced designs of exoskeletons in recent studies, some disadvantages remain.

Exosuits have recently drawn attention in the field of wearable devices owing to their clothing-like design. Made of fabric, an exosuit is lightweight and offers the advantage of a simple configuration, making it easy to wear; furthermore, it does not impose any constraints on the wearer’s movements [11]. Owing to the lightweight characteristics of exosuits compared with exoskeletons, they can reduce the metabolic penalty [12]. Given these advantages, exosuits can be considered as wearable devices that are suitable for use in daily life.

However, despite these advantages, exosuits have a shortcoming as wearable devices: they can be easily deformed when subjected to assistive forces, which leads to the delivery of an insufficient force to the target joint. Various studies have been conducted on mitigating the issue of deformation. Wehner et al. [13] used many of the anchoring points to reduce the deformation of the garment, thus reducing the loss of power (Figure 1C(e)). However, this method negates the existing advantages of exosuits, such as its lightweight, simple configuration and easy-to-wear characteristics, to some extent. As an early version, Asbeck et al. designed an ankle assistance exosuit that transmitted force by dissipating the supporting region from the pelvis to ankle in multiple directions to minimize suit deformation [6]. The state-of-the-art version of the ankle’s exosuit, shown in Figure 1C(f), mitigates the deformation problem by using additional clothing worn around the waist and special shoes with extra anchor points for support [14]. Although the stiffness of exosuits could be maximized as much as possible based on these designs, it needs other anchor points in addition to the assisted ankle, which increase the weight and complexity of the exosuit. The effect of weight on the body increases as it goes down from the waist to the thigh, knee, and ankle. Accordingly, reducing the weight and simplifying the structure of ankle assistance suits is a very important consideration [11]. Methods to prevent deformation can be classified into three categories. As can be seen in Figure 1A, the method through the addition of hard parts, and the method using both. Through this, the necessity of developing a soft and compact exosuit can be confirmed.

To mitigate the deformation concern in exosuits, we implemented a bio-inspired pattern methodology to the apparel design. Human gait evolved over a period of over >500,000 years since the time of Australopithecus. Over this period, our body structure acquired an efficient bipedal gait. If a suit design emulates this optimal body structure, it will have the potential to produce a suit with a lighter and more efficient form than that of the ones that are currently available. There have been many cases of wearable robots wherein limitations have been resolved by using biologically inspired methods. Asbeck et al. [6] implemented a lightweight and efficient exosuit whose design was based on an understanding of how humans walk. By mimicking the wearer’s skeletal structure and ankle joint parts of the biological limb, a more transparent, safe, and effective architectural design of the exosuit can be achieved. Park et al. [15] emulated the musculoskeletal system composed of muscles, tendons, and ligaments and provided sufficient fixation power to the suit without a rigid frame (Figure 1C(g)). Yang et al. developed a passive hip exosuit to improve the running efficiency based on the dynamic action of the hip’s flexor ligament during running [16]. Recently, studies that utilize biologically inspired methods are being conducted not only for exosuit design, but also for assistance strategies. Nuckols et al. [17] measured muscle dynamics during walking and developed an assistance strategy based on this as shown in Figure 1C(h). 

**Figure 5:**
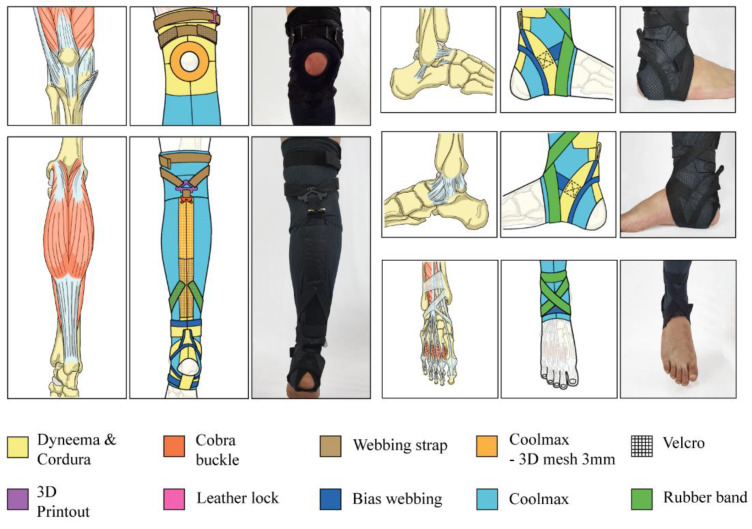
Parts of kneE-XOcks, designed by bio-inspired body anatomy, are made of eleven types of materials that ape the characteristics of body anatomy. As Dyneema and Cordura have high rigidity, they are used in parts that support and transmit the power of kneE-XOcks. Coolmax and 3D mesh have high breathability and cushioning, which increases the wearability of kneE-XOcks. Cobra buckle, leather lock, webbing strap, and bias webbing connect each element of kneE-XOcks to each other, allowing kneE-XOcks to be composed as a single garment. Unnecessary surplus in kneE-XOcks can be minimized using Velcro and rubber bands.

Our study ultimately aims to make the exosuit easy for anyone to use in a variety of environments. Our ankle exosuit design is called kneE-XOcks and is designed to have a similar appearance to knee socks in order to smoothly cover the anatomical regions from the knee to the foot (Figure 3). As a result, kneE-XOcks have a more compact and soft design than existing ankle exosuits as shown in Figure 1B. The design pattern of kneE-XOcks is based on the anatomy of muscles, ligaments, and tendons, and is placed at the ankle–foot complex and knee joint (Figure 4). In addition, by considering the differences of properties among the muscles, ligaments, and tendons, we apply a hybrid patterning technique that utilizes a combination of fabrics having varied characteristics (Figure 5). The hybrid patterning technique can mitigate deformation of the exosuit via lighter and simpler configurations compared with the existing ankle exosuits. Furthermore, the compact and low-profile design of kneE-XOcks enable them to be worn in conjunction with varied attire, such as pants or shoes (Figure 8). 

The rest of the paper is organized as follows. Section 2 introduces the human leg’s anatomy and the function of each region. Section 3 introduces the design patterns of kneE-XOcks, which was inspired by the human leg’s anatomy. Based on the introduced design patterns, Section 4 discusses the production of kneE-XOcks prototypes and specifically describes the fabric and procedure of production. Section 5 evaluates the performance and strengths of KneE-XOcks through the wearability, human-suit stiffness, and mobile actuation prototype tests. Finally, Section 6 compares the evaluated performance with other state-of-art exosuits to summarize the conclusions and contributions of the study.

## 2. Human Leg Anatomy and Functions

Human bipedal walking has evolved over several millennia. Consequently, an optimized anatomy has been developed for a bipedal gait that can transmit power efficiently within the musculoskeletal system composed of muscles, tendons, and ligaments, from the pelvis through the knee to the ankle [2]. The locomotor unit, which is composed of 11 joints of the pelvis and lower limb, occupies only approximately 30% of the human body mass but supports and moves the passenger unit corresponding to the remaining 70%. Furthermore, it performs various roles, such as propulsion, shock absorption, posture stability, and energy saving [18].

In humans, the main anatomical entities associated with the transfer of force are bones, skeletal muscles, tendons, ligaments, and retinacula. Their main function is to transfer the force generated through contraction of skeletal muscles to bones and other structures to make them move. Bones are connective tissues that form the majority of the skeleton. Muscles are tissues comprised of muscle cells that are responsible for movement and posture maintenance through contraction movements. Tendons allow muscles to attach to bone and have the characteristic of being very strong. Ligaments are connective tissues that connect bone to bone, provide joint stability or limit operability, and prevent certain actions. Finally, the retinaculum is responsible for fixing and stabilizing the tendons in their respective positions [19].

**Figure 2:**
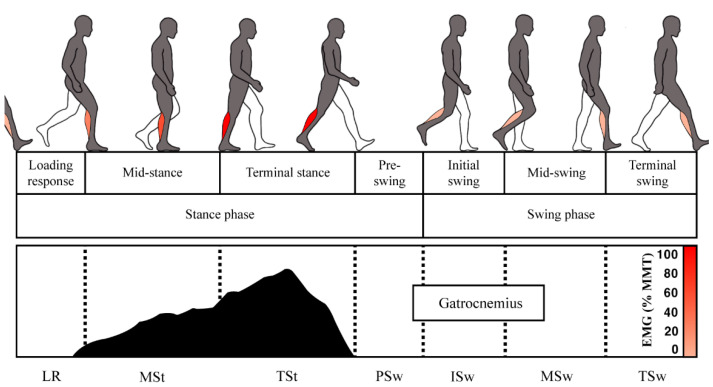
Muscle activity of gastrocnemius for each gait cycle section. Gastrocnemius is most activated in the section from terminal stance to pre-swing and plays a role in moving the body forward by performing plantar flexion.

We designed a new ankle exosuit inspired by these body elements. Each of these body elements was reproduced using fabrics and clothing subsidiary materials suitable for each characteristic. In particular, kneE-XOcks is inspired by the elements related to the gastrocnemius, one of the most active muscles in ankle movement. In human anatomy, while transitioning from the terminal stance phase to pre-swing, the gastrocnemius is activated to generate ankle plantarflexion movement, thus allowing the body to move forward (Figure 2) [20]. By studying the muscle, tendon, ligament functions, and bone structure during this gait cycle, we built a bio-inspired design pattern of kneE-XOcks that assists ankle joint movement (Figure 4). We theorized that emulating these constructions based on the human anatomy that has evolved over millions of years will result in a lightweight and simpler design of an ankle exosuit compared with other wearable devices.

## 3. Bio-Inspired Design Patterns for kneE-XOcks

### 3.1. Essential Characteristics for kneE-XOcks

First, as the ankle exosuit is meant for daily wear, it should be slim, compact, comfortable, and have a low-profile design [21]. Harvard University’s state-of-the-art ankle exosuit is considered the state-of-the-art design in terms of these features. However, it requires an additional waist belt to prevent the exosuit from slipping and provide an anchor point that can support the counteraction of assistive forces; this increases the volume of the design [14].

**Figure 3:**
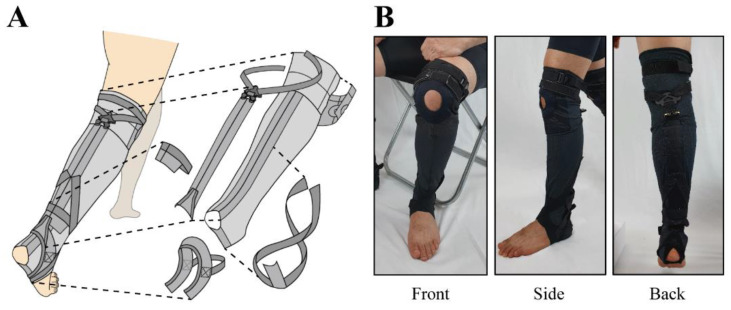
Overall composition of kneE-XOcks from the knee to the sole of the foot. (**A**) As each part is a separate piece of garment, the wearer can quickly and comfortably pull on kneE-XOcks as if they were wearing socks. (**B**) Consequently, kneE-XOcks can have a thinner and lighter design.

The overall structure becomes more complicated as the weight and volume increase, so this ankle exosuit becomes difficult to put on and take off easily. Consequently, it is onerous to use it in daily life [9]. To implement an ankle exosuit that can be worn casually in everyday life, we aim to eliminate the need for the additional waist belt so as to make the design slimmer and more compact. We selected the knee anchor point to replace the action of the waist anchor. In accordance with these changes, we developed a new kneE-XOcks design pattern (Figure 3).

Second, kneE-XOcks should not be deformed easily when assistive forces are applied, as the deformation can reduce force capability and cause a loss of force transmission. Specifically, kneE-XOcks should be designed so as to support the force transfer upward along the calf, at the ankle’s anchor point, thus surrounding the heel.

Simultaneously, it must not impede the movement of the wearer. As kneE-XOcks are designed as a shape that wraps around the ankle, the range-of-motion (ROM) of the ankle while the kneE-XOcks are worn is an important consideration for comfortable movement. In the case of ankle joints, the most important movements are plantar-/dorsiflexion in the sagittal plane, ab-/adduction in the transverse plane, and in-/eversion in the frontal plane. Based on the combination of these movements, the ankle produces three-dimensional body movements referred to as supination and pronation [22]. These movements occur within a constant ROM. On average, adult males aged 21 to 39 years have plantarflexion and dorsiflexion ranges of 35.3° and 38.1° in the sagittal plane, respectively. Furthermore, in the frontal plane, they have an inversion range of 34.6°, an eversion range of 37.5° [23], and an average ROM of 75.9 ± 4.1° (60.1°~107.7°) in the transverse plane [24]. Considering the range of these movements, the exosuit should be designed to allow free movement within these areas.

In summary, the required characteristics of kneE-XOcks are as follows. To maintain the advantages as an exosuit, it should be light and have a simple structure. Concurrently, for the efficient transmission of force, the deformation must be small. In addition, for easy use in daily life, there should be no restrictions on the attire that the user can wear, such as pants and shoes.

### 3.2. Bio-Inspired Methodology for Design Pattern

#### 3.2.1. Overall Concept of kneE-XOcks

**Figure 4:**
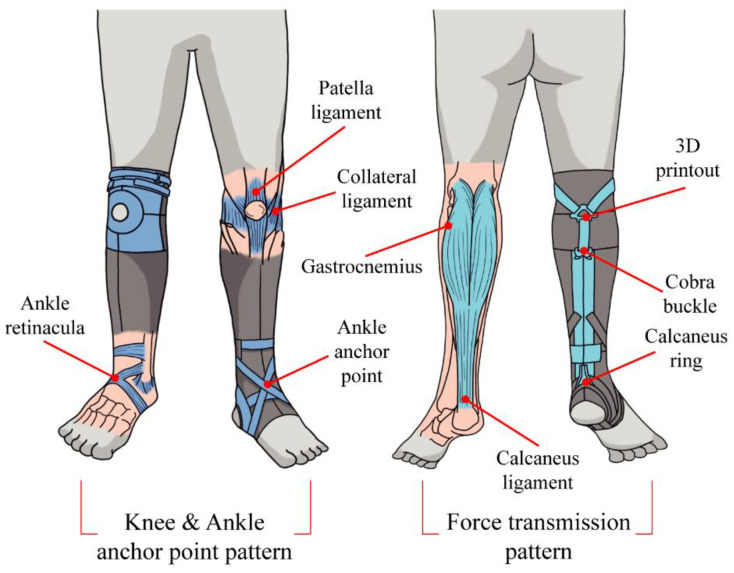
Anchor point pattern and force transmission pattern designed bio-inspired by the muscles, ligaments, and retinacula play a role in supporting and transmitting the force of the kneE-XOcks. The force transmission pattern mimics the function and shape of gastrocnemius and performs plantar flexion at the ankle joint. At this time, knee anchor point and ankle anchor point prevent the kneE-XOcks from being taken off and play a role in transmitting the force to each correct position. The knee anchor point mimics the function and shape of the ligaments around the patella, and the ankle anchor point simulates the function and shape of the ankle retinacula.

The kneE-XOcks are inspired by human anatomical parts (muscles, tendons, ligaments, and retinacula) involved in gait. Adopting a bio-inspired methodology, we aim to reproduce the functions of the human anatomy in achieving an efficient gait by mimicking the shapes, structures, and material characteristics of human anatomical structures. However, as it is not possible to mimic their shapes exactly, we can be inspired by their functions and use the components most suitable for the exosuit (Figure 4).

The overall design of the kneE-XOcks is a three-dimensional covering of body parts ranging from the knees to the soles of the feet and is shaped like knee socks or stockings (Figure 3). There are main three considerations of pattern designs, namely, the force transmission, anchor point, and clothing composition patterns. The force transmission pattern transmits the force of the suit, while the anchor point patterns help the force act at a proper position (Figure 4). Finally, careful textile selection serves to combine all elements of the kneE-XOcks as a single completed garment that can perform its role effectively. The bio-inspired components of the kneE-XOcks are mainly related to the transmission pattern design that transmits the assistive forces to enable exosuit operation as well as to the anchor point that allows these forces to act at the appropriate positions.

#### 3.2.2. Consideration of Force Transmission Pattern

The force transmission pattern part mimics the gastrocnemius muscles and relates to the generation of ankle plantarflexion moment. The gastrocnemius is the most superficial muscle in the posterior compartment and is also the largest muscle in the leg. It comprises a medial head, which is attached between the adductor tubercle and the surface of the medial condyle, and the lateral head, which is attached to the surface of the lateral femoral condyle. The two heads are joined together and connected to the calcaneal tendon attached to the heel (Figure 4). This shape of gastrocnemius is contracted by a tibial nerve to act as a plantarflexion at an angle point or bend the leg at a knee point [25]. The force transmission part starts from the posterior part of the knee and ends at the ankle along the calf and was manufactured in a Y-shape pattern (Figure 4). Owing to this bio-inspired shape, it is possible to effectively assist plantarflexion while transmitting the force from above to the ankle joint and allowing the knee to bend naturally.

#### 3.2.3. Consideration of Force Transmission Pattern

The roles of anchor points, including those of the knee and ankle anchor points, are essential for efficient operation of the force transmission pattern to directly imitate the gastrocnemius. Among the two anchor points, the knee anchor point is fixed around the knee, holds the downward pulling force during the walking motion, and prevents the suit from sliding off. The other anchor point, i.e., the ankle anchor point, is fixed around the sole and ankle of the foot, and thus prevents the removal of the suit by force.

The knee anchor point mimics the ligaments around the patella of the knee joint. The periphery of the patella consists of the patellar ligament and collateral ligaments. The patellar ligament is the continuation of the quadriceps femoris tendon inferior to the patella and the collateral ligaments; one exists on each side of the knee joint, which together stabilize the hinge-like motion of the knee [19]. By mimicking these ligaments surrounding the patella in an O-shaped pattern, a knee anchor point was designed to act as an anchor point without interfering with the bending of the knee (Figure 5). The anterior side of the knee anchor point, which receives directly the force generated from the force transmission pattern part, uses Dyneema fabric to minimize deformation. As the posterior side of the knee anchor point is a part wherein considerable contact between clothes and skin occurs as the knee is bent, sweat-wicking and quick-drying fabrics were used to increase the wearer’s convenience.

The ankle anchor point mimics the retinacula and ligaments of the ankle joint. Several pieces of bone in the ankle joint are fixed and stabilized by medial and lateral ligaments, while two extensor retinacula strap the tendons of the extensor muscles to the ankle region and prevent tendon bowing during the extension of the foot and toes. As such, the ankle joint has several ligaments and retinacula surrounding the ankle, like an ankle brace with a hole in the heel (Figure 5). The anchor point of the ankle, designed after this shape, is made of various materials that wrap the ankle smoothly so it can serve as an anchor point without interfering with the extensive movement that occurs in the ankle joint. As this anchor point must also have rigidity to withstand the upward pulling force generated by the force transmission part, the deformation was minimized by using Dyneema.

#### 3.2.4. Textile Selection Considerations

The pattern corresponding to each body part uses materials with the corresponding characteristics to harness the advantages and overcome the disadvantages. For example, the part covering the body shape mainly uses stretchable fabric with flexibility and elasticity to allow movement without hindering the muscle; conversely, it also uses a highly rigid fabric to increase the transfer of the force to the force transmission pattern part of the suit.

The ligament-mimicking part consists of highly stiff materials, such as straps, which serve as support; these are not easily deformed by muscle and force acting on the suit. Finally, the part imitating the tendon and retinaculum consists of a material such as an elastic rubber band or bias webbing, and it increases stability by connecting between highly rigid materials without hindering ankle movements (Figure 5).

### 3.3. Other Considerations in Apparel Design

As parts emulating human anatomical structures impact the wearability of the kneE-XOcks, it is necessary to connect each part as a garment, effectively enabling the functionality of each part and preventing the suit from being distorted or allowing any unintended movement. This consideration related to wearability consists of a base layer composed of soft and stretchable fabric to enhance the wearer’s convenience and a fixing layer made of fabric that is moderately soft but has high rigidity to secure the garment’s stiffness. Unlike other exosuits, which secure stability through a joint that extends to the hip, kneE-XOcks only comprises parts that extend from the knee to the ankle; thus, it is inevitably vulnerable to suit slippage effects. The fixing power of the suit is therefore secured by using two types of fixing layer made by straps or rubber bands. These types of fixing layers include the following: the knee fixing layer, wherein the strap surrounding the end of the femur and the patella is fixed using a leather lock, and the arch fixing layer, which covers the posterior part of the ankle from the arch of the foot, thus supporting the arch and preventing the suit from been unintentionally taken off, even following vigorous motion of the ankle. As the fixing layer contains Velcro, the size of the suit can be adjusted according to the wearer. They also reflect the curves of the human body via the draping method, thereby increasing the transfer of force generated by the suit without interfering with the body’s movement. Based on this, all parts of kneE-XOcks can have high degrees of completion by connecting them as single garments, while maintaining their individual characteristics.

## 4. Prototype Manufacturing of kneE-XOcks

### 4.1. Utilized Materials for Components

As the overall composition of kneE-XOcks is based on fabric, it is essential to use fabrics and subsidiary materials that match the characteristics of each part to enhance the advantages and mitigate the weaknesses. To this end, we synthesized kneE-XOcks using four types of fabrics, three types of subsidiary fabrics, and three types of subsidiary materials, each having unique characteristics. The fabrics included Dyneema, Cordura, Coolmax, and 3D-mesh. The subsidiary fabrics included rubber bands, webbing straps, and bias webbing. The subsidiary materials included cobra buckles, leather locks, and Velcro (Table 1).

Dyneema fabric, which is 15 times stronger than steel fiber of the same weight and 45% lighter than aramid fiber (mainly used for body armor, but more flexible), is a key fabric used in kneE-XOcks. As shown in Figure 5, Dyneema was used with webbing or cobra buckles in places where strong support is required, such as at the force transmission pattern and anchor point parts.

However, because Dyneema has several limitations, it must be appropriately combined with fabrics that can play an auxiliary role. Dyneema has rough and stiff surfaces, which may cause discomfort when in direct contact with the wearer’s skin. Therefore, Cordura fabric, which is wearer-friendly compared to Dyneema and has a certain degree of rigidity, high-moisture permeability, and drying speed, was used as a lining for the area beneath the Dyneema fabric. In addition, a mesh pad made of 3D structure was attached to the inner parts of the Dyneema fabric (i.e., the force transmission pattern part) to prevent the skin from being chafed by the movement of the wire caused by the driving of the motor.

Furthermore, in the bifurcated piece of the transmission pattern part, webbing straps with Velcro were attached to minimize the errors in the position of force transmission or the degree of tightness of clothing owing to differences in body size such as knee circumference or thigh circumference for each wearer. By using a webbing strap, the wearer can easily adjust the size without external help, which would be difficult to do if it was only made of Dyneema.

While Dyneema fabric acts as a skeleton for kneE-XOcks, Coolmax fabric acts as the flesh that connects each part. The Coolmax fabric is composed of knit-weaving with good elasticity and is made of fibers with a wide cross-sectional area. Thus, it provides comfort to the wearer while also being quick-drying. The Coolmax fabric used for the base layer of kneE-XOcks enhances the completeness of the kneE-XOcks.

Finally, the elastic rubber band constitutes a fixing layer to reduce the deformation of clothes and prevent unintentional slipping. This fixing layer was also attached with Velcro or a leather lock, so the wearer can easily control the degree of pressure by adjusting the tension (Figure 5).

### 4.2. Manufacturing Process

**Figure 6:**
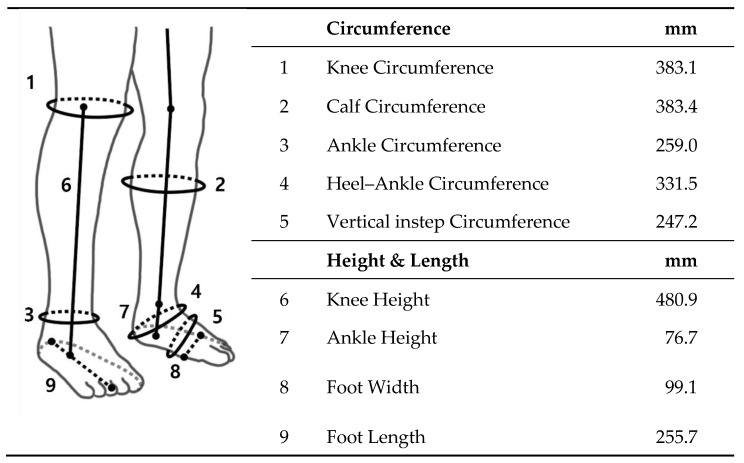
As kneE-XOcks is manufactured with a tight design that attaches to the body, it is necessary to use the precise actual size to maximize the wearability and auxiliary effects of kneE-XOcks. In order to reflect the body type of the main subjects in their 20 s and 30 s, the size of Korean adult males aged 20 to 39 years (measured by Size Korea) was referenced.

KneE-XOcks is a soft exosuit that can be worn as an everyday wear garment. The first part of the manufacturing process of the kneE-XOcks is to take the overall shape of the base layer from knee to foot. As the shape of the body from knee to foot is one of the sections with the largest ROM and the most flexion, there is a limit in ensuring that the base layer wraps smoothly around the body like socks and in making it easy to put them on and take them off by using only two-dimensional (2D) apparel pattern making. To mitigate this concern, the shape of the base layer was produced with the use of the draping pattern method, while directly putting the appropriate fabric for each part on the body of mannequin. Based on this, the 3D curve of the body can be reflected in the 2D apparel pattern. The body size used at this suit refers to the average size of men aged 20–39 in Korea (Figure 6) [23].

The body size required for the production of the base layer is largely divided into circumference and height. There are five types of circumference types: the knee circumference passing through the mid-point of the kneecap, the calf circumference passing through the calf protrusion point, the ankle circumference passing through the ankle point, the heel-ankle circumference passing through the bottom part of the heel and ankle point, and the vertical instep circumference passing through the instep. There are four types of height and length: the knee height from the floor to the top of the shinbone, the ankle height from the floor to the ankle bone, the foot width, i.e., the horizontal distance between the point on the inside of the foot and the point on the side of the foot, and the foot length from heel to toe. To proceed with draping by applying these dimensions, it is necessary to calibrate the mannequin according to each dimension using additional pads or fabric. With the use of this mannequin, we created a base layer that fits the average size of men aged 20–39 in Korea [23].

**Figure 7:**
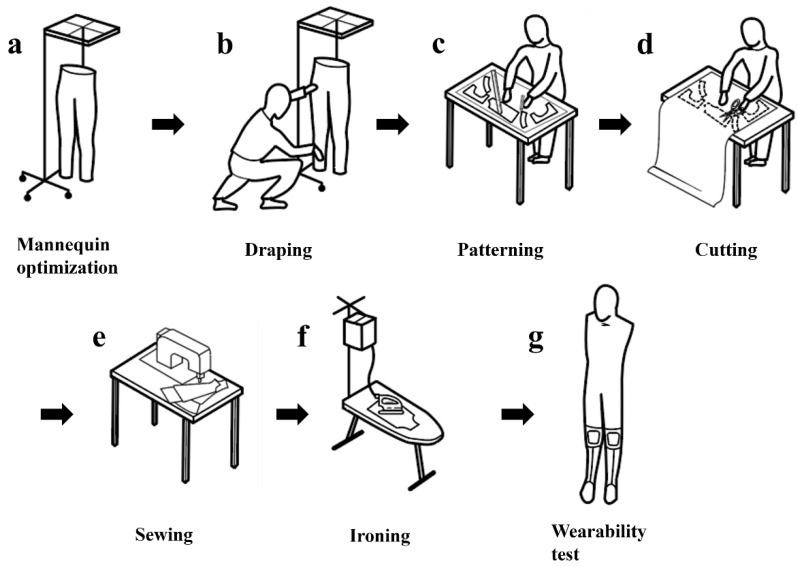
kneE-XOcks was manufactured through a seven-step manufacturing process. After optimizing the mannequin to fit the target dimensions, each pattern is created using the draping method. After converting the three-dimensional pattern obtained through this process into a flat pattern, pieces of fabric suitable for each pattern are cut out. After sewing these and connecting them into one garment, finishing work such as ironing is performed. Finally, the kneE-XOcks are completed by reflecting the feedback obtained through the wearability test.

The kneE-XOcks is manufactured through the steps shown in Figure 7. After optimizing the mannequin by substituting the size of Figure 6 into mannequin, Based on this size, a base layer made of Coolmax fabric was produced. After the base layer was completed, the force transmission pattern was manufactured using the bio-inspired design of the gastrocnemius muscle. Each section of the part was placed in the corresponding position of the muscle to effectively emulate the function of gastrocnemius. The straps acting as the medial and lateral heads of the gastrocnemius were gathered together based on the triangular 3D printout, and the gathered straps fixed the cobra buckle, which held the wire to be operated through the actuator. Through a rectangular piece made of Dyneema, the cobra buckle and the ring fixed at the calcaneus position were connected, and a pad made of 3D mesh material was added inside to prevent the wearer from getting injured owing to the friction caused by the wire (Figure 5).

Based on the position of the force transmission pattern part, the knee’s anchor point was located in the upper position, and the ankle’s anchor point was located in the lower position. In this manner, as shown in Figure 3a, the base layer, anchor points, and force transmission parts are connected. After all parts of kneE-XOcks are connected, additional fixing layers are sewn to prevent deformation of the suit or unintentional slipping owing to body motion or actuator forces. As kneE-XOcks was mostly made of fabric, there were bound to be creases of folds in the previous process, so the kneE-XOcks were ironed to straighten these parts and give them a proper shape. Finally, the kneE-XOcks can be completed by applying the feedback obtained through the wear test and making final modifications.

### 4.3. Manufactured Prototype

The weight of the suit of the kneE-XOcks with subsidiary materials, excluding the actuator and wire, was 106.3 g. Of the weight of the kneE-XOcks, the force transmission pattern accounts for 48 g, and the anchor point accounts for 40 g. kneE-XOcks can be worn at once by inserting the wearer’s foot, just like wearing socks. After wearing the kneE-XOcks, it must be ensured that the ankle anchor point (starting from the arch of the foot) is in the correct position, and then the size must be adjusted and fixed using Velcro. Subsequently, Velcro must be used on the top of the knee and on the calf to remove unnecessary slack, and the knee’s anchor point must be adjusted to fit the wearer perfectly.

Using a sewing method suitable for each part is another way to maximize the advantages of kneE-XOcks. When sewing kneE-XOcks, overlock and cover stitches were mainly used to withstand stronger tension than when fabricated using general sewing and to prevent easy tearing. In addition, the long seam below the knee anchor point based on this sewing method serves as a reference check line irrespective of whether the garment is warped or not and ensures that it is worn stably along the shinbone (Figure 3b).

## 5. Results and Discussion of the kneE-XOcks

### 5.1. Wearability Test

**Figure 8:**
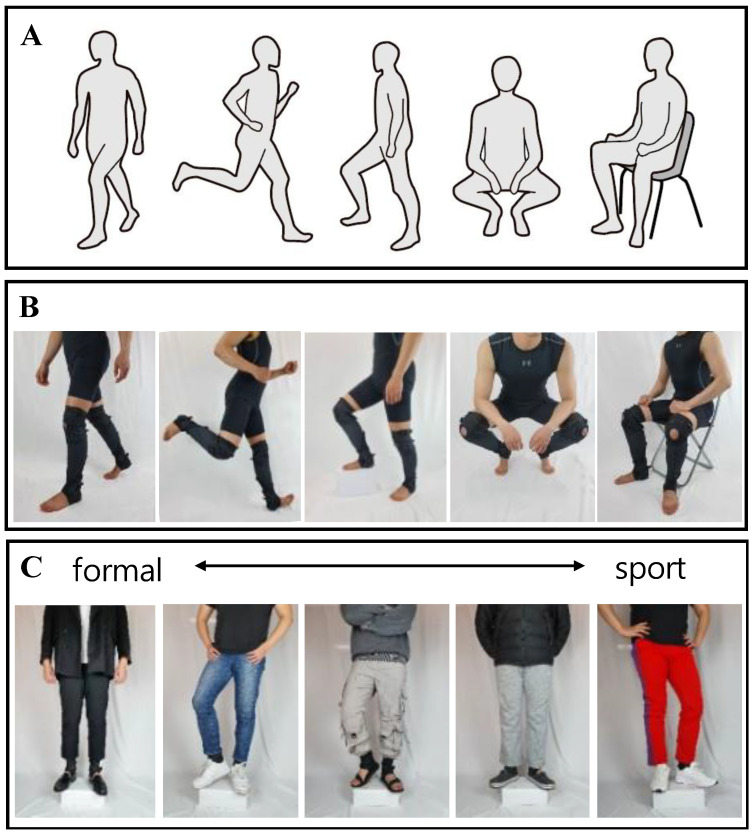
(**A**) Humans perform various movements in daily life. The lighter and simpler design of kneE-XOcks can serve as an immense advantage in everyday life. (**B**) Various movement performance tests and (**C**) various types of clothing tests demonstrate that wearing kneE-XOcks does not interfere with daily movement performance and is not restricted by any type of clothing.

The new kneE-XOcks had a weight of 106.3 g (without wires and sensors). In addition, the volume was reduced noticeably by eliminating the additional parts that existed above the knee in the design that allowed anchorage only at the knee and ankle. Therefore, it does not differ much from the thickness and volume of daily socks, befitting the name kneE-XOcks, a name inspired by a type of socks known as knee socks (Figure 8). The time taken to put on the suit, excluding the driving part, was approximately 30 s per side on average. Therefore, the seven subjects who wore kneE-XOcks for the first time were able to put it on and take it off in a similar manner to wearing/removing socks without external wearing assistance.

The advantages of kneE-XOcks are not limited to being lightweight and allowing fast wearability. As shown in Figure 8, various movements necessary for daily life can be performed without difficulty, and there are almost no restrictions on wearing everyday clothes and shoes. We conducted wearability tests with kneE-XOcks on seven men aged 25.8 ± 1 years with various body sizes (height: 176 ± 5, weight: 71.9 ± 6.8). As shown in Figure 8, the kneE-XOcks motion performance was evaluated while various movements were performed. Based on this, it can be observed that even if kneE-XOcks are worn, there are no major difficulties in performing daily activities.

### 5.2. Human-Suit Series Stiffness

We synthesized an ankle exosuit that is easier to wear and lighter than the existing ankle assistive exosuits, but has a similar level of stiffness. To verify this, a series of stiffness tests was conducted on the human suit. Owing to having a soft material-oriented composition, a stiffness model is essential to compensate for the nonlinear deformation that occurs when the motor force is transmitted to the kneE-XOcks [26].

**Figure 9:**
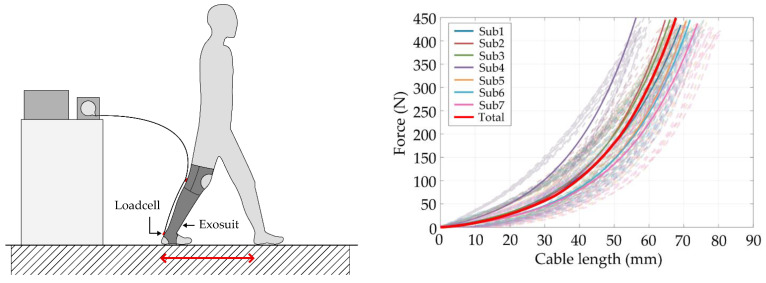
As an ankle exosuit, the most important function of kneE-XOcks is its ability to transmit power to the body. A stiffness test in a static posture shows that kneE-XOcks can transmit force to the body without significant deformation.

To obtain this, a stiffness test as shown in Figure 9 was conducted on seven subjects (age: 25.8 ± 1, height: 176 ± 5, weight: 71.9 ± 6.8) who wore kneE-XOcks, and the relationship between the changed cable length and cable force value at this time was analyzed to measure stiffness in a static posture. All subjects were informed about the contents of the study, including the purpose, the protocols, and inclusion/exclusion criteria, and consented prior to participating in the study. Protocols in the study were approved by the Chung-Ang University Institutional Review Board(1041078-202107-HR-214-01C).

The posture was set as close as possible to the time at which the assistive motion of kneE-XOcks actually occurs, based on the time point at which the terminal stance leads to the pre-swing. This corresponds to 31–50% of the gait cycle and belongs to the range in which gastrocnemius is maximally activated [20].

In addition, each subject’s feet were placed apart as the stride length of the subject’s feet (average preferred walking speed of subjects: 1.715 m/s, average preferred stride length of subjects: 0.802 m) based on the results of measuring each subject’s preferred walking speed and preferred stride length. In the case of preferred walking speed and stride length, measurements were conducted on flat ground at a distance of 40 m, and the values measured for the 20-m section, excluding the 10-m front and rear acceleration and deceleration sections, were used.

In this static posture, a motor (RE 50, 200W, Maxon, Sachseln, Switzerland) coupled with a gearhead (GP 62, Maxon, Sachseln, Switzerland) was used to transmit force to the kneE-XOcks, and a machined pulley was connected to this motor. A Bowden cable (ALLIGATOR 31-STRAND INNER CABLE, ALLIGATOR, Changhua City, Taiwan) was used with cable housing BHL100, JAGWIRE, Changhua City, Taiwan) and was connected to the pulley, and the power of the motor was transmitted through this cable. The motor was controlled by a motor driver (EPOS 4 70/15, Maxon, Sachseln, Switzerland), and the force was measured using a loadcell (LSB 205, Futek, Irvine, CA, USA). The control system was designed by using a system design platform (LABVIEW, National Instruments, Austin, TX, USA).

Figure 9 shows the result of the stiffness evaluation obtained under the above conditions, i.e., the stiffness diagram when a force of up to 450 N is repeatedly applied ten times to seven subjects wearing kneE-XOcks. The model of human-suit stiffness was fitted based on the fitting equation proposed by G Lee et al. [26].

δ_suit_ = C_1_ ln(C_2_F + 1) (1)

where the C_1_ and C_2_ are the coefficients of the human-suit stiffness model, F is the cable force, and δ_suit_ is the cable displacement.

When comparing only absolute stiffness values based on a force of 375 N, which is the highest value in the stiffness measurement value of the ankle exosuit of Bae et al. [14], the average stiffness value of kneE-XOcks is 21% lower than that of Bae et al. However, when this is converted into a stiffness value for suit-weight and compared, it shows that it has about five times stronger stiffness.

### 5.3. Preliminary Mobile Test of the kneE-XOcks

To verify the actual force transmission performance of kneE-XOcks in a walking situation and to measure concurrently the completeness as a garment in a situation where the power of the motor acts on the suit, a preliminary mobile test on the treadmill wearing the kneE-XOcks with customized mobile actuator was conducted. Subjects who yielded the highest results in the stiffness test with the human-suit were selected.

**Figure 10:**
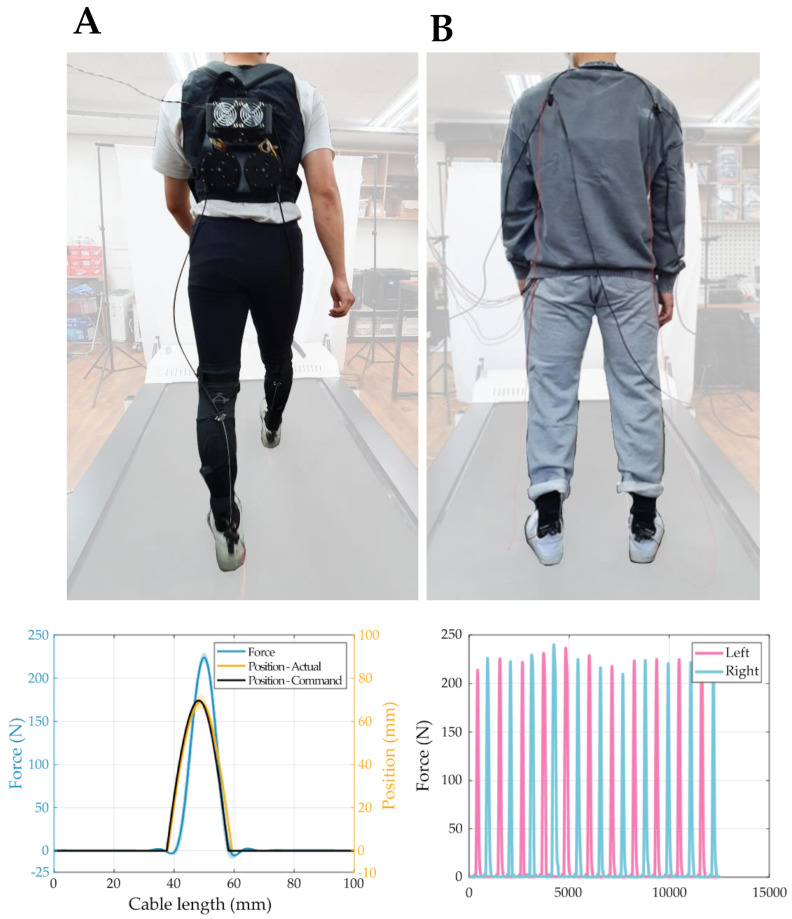
(**A**) A walking test through a combination with a mobile actuator can demonstrate the advantages of the lighter and simpler design of kneE-XOcks. (**B**) It can be worn under everyday clothes and driven without difficulty. From the result graph, it can be seen that the kneE-XOcks can operate without any issues as an ankle exosuit even when combined with a mobile actuator.

These subjects (age: 26 years, height: 175, weight: 73) wore kneE-XOcks and then performed a walking test at the subject’s preferred walking speed on a treadmill (1.25 m/s). The test was conducted through a mobile system, as shown in Figure 10. The motor (PEQDD) which coupled with a gearhead (GP 32, Maxon, Sachseln, Switzerland) was used to provide force to the kneE-XOcks. A pulley was linked to the motor. The Bowden cable (ALLIGATOR 31-STRAND INNER CABLE, ALLIGATOR, Changhua City, Taiwan) was used with cable housing (BHL100, JAGWIRE, Changhua City, Taiwan) and was connected to the pulley that transmits force.

The motor operated according to the position control method based on the profile of Lee et al. [26], and the entire control system of the test was a real-time controller (CompactRIO, National Instruments, Austin, TX, USA) and a system design platform (LABVIEW, National Instruments, Austin, TX, USA). The motor was controlled through EtherCAT Communication using a motor driver (Gold Solo Twitter 10/100, Elmo Motion Control, Petah Tikva, Israel), and the force transmitted to kneE-XOcks was measured using a loadcell (LSB 205, Futek, Irvine, CA, USA) via analog communications.

The total weight of the actuator system used in the test was 2.47 kg. Of the total weight, the cables and sensors used to transmit and control the assistive force weigh 226 g. Our research group developed the actuation system ourselves, and we plan to reduce the volume and weight through further development and advancement.

kneE-XOcks has the advantage of being lighter and simpler than existing exosuits, and this advantage can be best demonstrated by conducting a walking test through a combination with a mobile actuator. The results of the previous walking test show that kneE-XOcks can operate without lethal deformation even when the wearer is moving, and that they can be combined with mobile actuators without any problems (Supplementary Video S1).

## 6. Conclusions

In this study, kneE-XOcks, a new type of ankle exosuit, was created by adopting a design method based on a bio-inspired pattern, and the improved stiffness-to-weight ratio compared with existing ankle exosuits was verified. Based on the human anatomy, the bio-inspired pattern and manufacturing method effectively reduced the weight and volume of kneE-XOcks compared with existing ankle exosuits in order to increase the wearability while maintaining the assistive effect of an exosuit. We believe that the bio-inspired pattern and manufacturing method used in kneE-XOcks can serve as a “new exosuit design paradigm” to enable the research of new wearable robots using various assistive methods.

The performance test and verification of kneE-XOcks in this study were conducted using a tethered system. As shown in Figure 10, based on a combination with the mobile actuation system, kneE-XOcks’ goal of producing an easy-to-wear ankle exosuit resembling innerwear was achieved. If further research is conducted on the optimization with this mobile system, such as the lighter kneE-XOcks synthesized with a simpler design, it will be possible to manufacture an exosuit for use in daily life that (a) does not degrade the assistive performance and (b) does not have the restrictions of wearable robots. Moreover, since wearable robots are directly connected to the human body, it is essential to consider ergonomics [27,28]. The bio-inspired pattern, the method used to design kneE-XOcks, is closely related to these ergonomic studies. Therefore, we expect that the study results will emphasize the importance of ergonomic points of view in the field of exosuit.

As this study mainly deals with the conceptual proposal and verification study of a bio-inspired pattern, a detailed analysis on the efficacy of bio-inspired patterns should be conducted in the future through methods such as force relation analysis or finite element analysis. In addition, to validate the effect of KneE-XOck on the wearer, we plan to validate the performance of the KneE-XOcks by measuring the human response changes that occur during gait through kinematic analysis, EMG, and metabolic cost. By these means, it will be possible to identify and overcome the limitations of the current bio-inspired pattern, while simultaneously applying the bio-inspired patterning technique to other parts of the body to optimize each part of the body. The application of the bio-inspired patterning technique optimized for each exosuit that supports the various movements of various parts will effectively overcome the low usability of exosuits in daily life, which has been the exclusive domain of wearable robots thus far.

## Figures and Tables

**Table 1:** For each pattern of kneE-XOcks, fabrics and materials that match the characteristics of the body anatomy targeted for the bio-inspired pattern design were used. High stiffness fabrics and materials such as Dyneema and Cordura or webbing are used for force transmitting and supporting patterns. Fabric and material such as Coolmax with high breathability or rubber band were used for patterns that consider the wearer’s convenience.

	DYNEEMA	CORDURA	COOLMAX	3D-MESH 3 mm	RUBBER BAND	STRAP WEBBING	BIAS WEBBING
	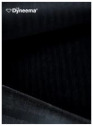	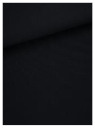	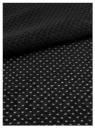	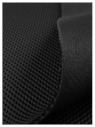	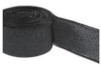	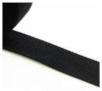	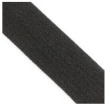
Fabric weight	109 g/m^2^	121 g/m^2^	136 g/m^2^	755 g/m^2^	927 g/m^2^	766 g/m^2^	371 g/m^2^
Tensile strength	Wale: 1800 N/5 cm Course: 1600 N/5 cm	Wale: 1500 N/5 cm Course: 910 N/5 cm	Wale: 330 N/5 cm Course: 210 N/5 cm	Wale: 1300 N/5 cm Course: 1100 N/5 cm	1300 N/5 cm	2700 N/5 cm	1800 N/5 cm
Tearing strength	Wale: 49 N Course: 72 N	Wale: 73 N Course: 49 N	Wale: 25 N Course: 15 N	Wale: 190 N Course: 180 N	N/A	N/A	N/A
Bursting strength	No burst (max 2060 kPa)	1700 kPa	530 kPa	No burst (max 2060 kPa)	N/A	N/A	N/A
Abrasion resistance	Over 20,000 Rubs	Over 20,000 Rubs	Over 20,000 Rubs	Over 20,000 Rubs	N/A	N/A	N/A
Water Vapor transmission	Under 30 g/m^2^	1044 g/m^2^	1524 g/m^2^	844 g/m^2^	N/A	N/A	N/A
Absorbency	Over 60 s	Over 60 s	1.0 s	1.0 s	N/A	N/A	N/A
100% Dry time	N/A	N/A	60.7 min	39 min	N/A	N/A	N/A

## Data Availability

Not applicable.

## Supplementary Materials

The following supporting information can be downloaded at: https://www.mdpi.com/article/10.3390/biomimetics7040148/s1, Video S1: Preliminary test of the kneE-XOcks with mobile actuation system.
